# Long‐Term Post‐Stroke Cognition in Patients With Minor Ischemic Stroke is Related to Tract‐Based Disconnection Induced by White Matter Hyperintensities

**DOI:** 10.1002/hbm.70138

**Published:** 2025-01-27

**Authors:** Renaud Lopes, Grégory Kuchcinski, Thibaut Dondaine, Loïc Duron, Anne‐Marie Mendyk, Hilde Hénon, Charlotte Cordonnier, Jean‐Pierre Pruvo, Régis Bordet, Xavier Leclerc

**Affiliations:** ^1^ U1172 – LilNCog (Lille Neuroscience & Cognition) Univ. Lille, Inserm, CHU Lille Lille France; ^2^ US 41 – UAR 2014 – PLBS Univ. Lille, CNRS, Inserm, CHU Lille, Institut Pasteur de Lille Lille France; ^3^ Department of Nuclear Medicine CHU Lille Lille France; ^4^ Department of Neuroradiology CHU Lille Lille France; ^5^ Department of Neuroradiology Alphonse de Rothschild Foundation Hospital Paris France; ^6^ Faculté de Médecine Université de Paris, PARCC, INSERM Paris France; ^7^ Department of Neurology CHU Lille Lille France; ^8^ Department of Pharmacology CHU Lille Lille France

**Keywords:** Bayesian model, cognitive impairment, ischemic stroke, tract‐based disconnection, white matter hyperintensities

## Abstract

Over a third of minor stroke patients experience post‐stroke cognitive impairment (PSCI), but no validated tools exist to identify at‐risk patients early. This study investigated whether disconnection features derived from infarcts and white matter hyperintensities (WMH) could serve as markers for short‐ and long‐term cognitive decline in first‐ever minor ischemic stroke patients. First‐ever minor ischemic stroke patients (NIHSS ≤ 7) were prospectively followed at 72‐h, 6 months, and 36 months post‐stroke with cognitive tests and brain MRI. Infarct and WMH volumes were semi‐automatically assessed on DWI and FLAIR sequences. Bayesian tract‐based disconnection models estimated remote pathological effects of infarcts and WMH. Associations between disconnection features and cognitive outcomes were analyzed using canonical correlation analyses, adjusted for age, education, and multiple comparisons. Among 105 patients (31% female, mean age 63 ± 12 years), infarct volume averaged 10.28 ± 17.10 cm^3^ and predominantly involved the middle cerebral artery territory (83%). WMH burden was higher in frontal periventricular white matter. Infarct‐based features did not significantly relate to PCSI. However, a WMH‐derived disconnection factor, involving commissural and frontal tracts, and the right superior longitudinal fasciculus, was significantly associated with PSCI at 6 months (OR = 9.96, *p* value = 0.02) and 36 months (OR = 12.27, *p* value = 0.006), particularly in executive/attention, language, and visuospatial domains. This factor, unrelated to WMH volume, outperformed demographic and clinical predictors of PSCI. WMH‐induced disconnection may be associated with short‐ and long‐term PSCI in minor stroke. Routine MR‐derived features could identify at‐risk patients for rehabilitation trials.

## Graphical abstract

No relationship was observed between infarct‐based features and post‐stroke cognitive impairment (PSCI). A Bayesian model illustrating stroke participants, latent factors, and tract‐based disconnection induced by white matter hyperintensities was associated with 6‐ and 36‐month PSCI. The model can be easily implemented in clinical practice as it solely relies on conventional MR sequences.

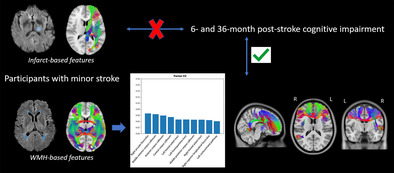

**Summary**

- The study did not find a significant relationship between infarct‐based features and 6 and 36‐month post‐stroke cognitive impairment (PSCI) potentially due to the methodological limitations in infarct location categorization.
- A Bayesian model based on tract‐based disconnection induced by white matter hyperintensities (WMH) was significantly associated with 6‐ and 36‐month PSCI, particularly in the domains of executive function, attention, language, and visuospatial abilities.
- The Bayesian model, based on conventional MR sequences, has the potential to be integrated into clinical practice. With further development, it could aid in identifying patients at high risk of long‐term cognitive decline after stroke.

**Abbreviations**
CCA: canonical correlation analysis
LDA: latent Dirichlet allocation
MNI: Montreal Neurological Institute
NIHSS: National Institutes of Health Stroke Scale
PSCI: post‐stroke cognitive impairment
WMH: white matter hyperintensities

## 1 Introduction

Over a third of stroke patients will suffer from post‐stroke cognitive impairment (PSCI) but there are no validated tools to clearly identify at‐risk patients in the early phase. In recent years, studies have explored clinical and imaging predictors for PSCI. Systematic reviews, such as those by Pendlebury and Rothwell (2009) and Filler, Georgakis, and Dichgans (2024), have synthesized evidence on risk factors, including acute infarct characteristics and white matter hyperintensities (WMH). Furthermore, a recently published predictor model by Weaver et al. (2021) highlighted the significance of acute infarct location in predicting PSCI, focusing on the maximum overlap of lesions (a topological approach).

In contrast to the topological perspective, the hodological approach considers the structural pathways traversed by the infarct. Disruption of these pathways, known as structural disconnection, can impair network efficiency, potentially leading to cognitive deficits (Catani and Mesulam 2008). Increasing evidence links PCSI to structural disconnection caused by infracts, which disrupt the connectivity of critical brain networks (Griffis et al. 2019).

WMH, frequently observed on brain MRI in stroke patients, are also an independent risk factor for cognitive impairment (Sivakumar et al. 2017). They may also disrupt white matter fiber bundles, affecting network integrity beyond the lesion site. Although the histopathological substrate of WMH is heterogeneous, with varying degrees of myelin and axonal loss (Prins and Scheltens 2015), their presence is widely recognized as indicative of chronic cerebrovascular injury. Diffusion‐weighted imaging (DWI) studies have shown that the microstructural changes within WMH correlate with axonal loss and demyelination, supporting their role as markers of ongoing white matter damage (Sun et al. 2006). Furthermore, WMH are associated with cardiovascular risk factors, such as hypertension and diabetes, which contributes to the development of WMH (Dickie et al. 2016; Williamson et al. 2018). These cardiovascular factors increase the risk of brain ischemic damage, and WMH are an independent predictors of future stroke risk (Ghaznawi et al. 2021).

Similarly, in a study of 319 stroke patients, Etherton et al. (2021) demonstrated increased axial and radial diffusivity (RD) in WMH compared to normal‐appearing white matter (NAWM), with axial diffusivity (AD), an indicator of axonal loss, serving as an independent predictor of WMH volume in acute ischemic stroke. Moreover, the presence of WMH is associated with widespread macro‐ and microstrutural changes in both gray and white matter, further highlighting their role in chronic cerebrovascular pathology (Brugulat‐Serrat et al. 2020; Vangberg, Eikenes, and Håberg 2019). One hypothesis is that the white matter tracts passing through the lesions will affect the connected structures (Aribisala et al. 2013). Despite these advances and the high prevalence of PSCI ranging from 30% to 55% in minor strokes (Lopes et al. 2021; Zhao et al. 2021), predicting PSCI remains challenging, where the heterogeneity of infarcts and WMH location complicates the use of traditional imaging markers. Our study aims to address this limitation by exploring WMH‐induced disconnection as a novel marker for PSCI.

We focused on MRI features from standard MRI sequences routinely used in clinical practice for the assessment of stroke patients, such as DWI to evaluate acute infarcts and Fluid‐Attenuated Inversion Recovery (FLAIR) to assess WMH. Structural disconnections were quantified by analyzing white matter fiber bundles directly involved by infarcts or WMH through the concept of structural connectivity (Duering et al. 2015). The development of large databases of diffusion MRI data and the analysis of structural connections among brain regions that are common in a large group of healthy subjects have allowed the construction of so‐called connectome and tractographic atlases (Yeh et al. 2018). Disconnectome approach estimates structural disconnection from lesion mask and tractographic atlas, by measuring the probability of normal white matter tracts passing through the lesion (Griffis et al. 2021). Approaches using white matter tract disconnection from infarct have been used to investigate brain network dysfunction, behavioral, and cognitive deficits after stroke (Griffis et al. 2019; Kuceyeski et al. 2016; Salvalaggio et al. 2020). No study has investigated the relationships between PSCI and white matter tract disconnection induced by WMH.

Both infarcts and WMH can alter multiple white matter tracts and are associated with cognitive impairment in different and/or multiple domains. Thus, the interaction between WMH and acute ischemic event can trigger a series of pathological events that lead to heterogenous trajectories of cognitive decline (Levine et al. 2015). To address this complexity, dimensional methods such as factor analysis (Blei, Ng, and Jordan 2003) can be employed to identify patterns of observable and latent anomalies at the patient level. This approach enables the possibility that multiple latent factors of white matter tracts disconnection can be expressed to varying degrees within a patient.

The objective of this study was to identify latent disconnection factors from infarcts and WMH in first‐ever minor ischemic stroke patients using latent Dirichlet allocation (LDA) and to determine whether these factors could be potential markers for short‐ and long‐term cognitive impairment. We defined minor stroke as a first‐ever ischemic stroke with a National Institutes of Health Stroke Scale (NIHSS) score of 7 or less at the time of admission. Additionally, we compared our approach with global and regional analyses of lesion volume and location, hypothesizing that PSCI could be explained by a combination of white matter disconnection due to infarct and/or WMH, accounting for variations in multidomain cognitive decline among stroke patients.

## 2 Materials and Methods

### 2.1 Study Population

Between 2010 and 2020, a total of 202 patients with first‐ever ischemic stroke were enrolled in the Study of Factors Influencing Poststroke Dementia cohort (STROKDEM; NCT01330160). Participants aged 18 years and older were included, while patients with the following criteria were excluded: (1) pre‐stroke dementia (defined as a short form Informant Questionnaire of Cognitive Decline in the Elderly [IQCODE] score of 64 or more) (Jorm 1994), (2) moderate or severe stroke (NIHSS score > 7), (3) no MRI‐visible lesion, (4) secondary hemorrhage, with type 2 parenchymal hematoma (Fiorelli et al. 1999), (5) MRI contraindications, (6) inability to speak, and (7) a poor understanding of the French language. The STROKDEM study conducted follow‐up assessments of the participants’ clinical, neuropsychological, and imaging from 72 h to 36 months post‐stroke.

### 2.2 Neuropsychological Assessment

The participants’ cognitive functions, including executive functions/attention, memory, language, and visuospatial abilities, were assessed 6‐ and 36‐month post‐stroke using psychometric data collected during a standardized neuropsychological examination (T.D.) (see Data S1). For each patient, we calculated test‐specific *z* scores based on published norms, corrected for age, gender, and education. We then obtained summary *z* scores by domain by averaging the test‐specific *z* scores in each domain. A summary *z* score ≤ 1.5 in at least one domain was used to determine PSCI (Lo et al. 2019; Zietemann et al. 2018). The PSCI diagnosis is determined at a multidisciplinary staff meeting attended by neurologists and neuropsychologists at 6‐ and 36‐month post‐stroke, ensuring that both objective measures and clinical judgment are used (Sachdev et al. 2014). This team ensures consistency between any subjective complaint by the patient or the principal informant and symptoms during clinical examination, and the neuropsychological assessment.

### 2.3 MRI Acquisitions

All participants underwent structural brain MRI after reperfusion therapy, if required, within 72 h of admission to the hospital using one 3T MRI scanner (Achieva, Philips, Best, the Netherlands) with a 16‐channel neurovascular coil. The imaging protocol notably included a 3D T1‐weighted (T1W) sequence (TFE sequence, repetition time: 9.84 ms; echo time: 4.60 ms; voxel size: 1 mm^3^; matrix size: 256 × 256 × 160 voxels, flip angle: 8°, inversion time: 697 ms), a diffusion‐weighted imaging (DWI) sequence (repetition time: 9000 ms; echo time: 76 ms; voxel size: 1.9 × 1.9 × 4 mm; matrix size: 128 × 128 × 38; one b0 and a *b*‐value of 1000 s/mm^2^ with one gradient direction in each of the three planes), and a fluid‐attenuated inversion recovery (FLAIR) sequence (repetition time: 9000 ms; echo time: 142 ms; voxel size: 0.65 × 0.97 × 4 mm; matrix size: 352 × 186 × 45, inversion time: 2800 ms).

### 2.4 Infarcts and WMH Mapping

Infarcts were manually delineated as hyperintense areas on DWI (G.K.). WMH were automatically segmented on FLAIR images using volBrain software (https://volbrain.net), and were manually corrected to exclude the acute infarct area (G.K.). Infarcts and WMH masks were normalized in Montreal Neurological Institute (MNI) space using linear and nonlinear transformations for the registrations of DWI/FLAIR images to T1W space and T1W space to MNI space, respectively (stnava.github.io/ANTs/). MNI152 template at 1 mm resolution were used (fsl.fmrib.ox.ac.uk/fsl/fslwiki/Atlases).

### 2.5 Infarcts and WMH Location

A vascular territory template in MNI space was used to create an incidence map of infarct location (Schirmer et al. 2019). The template was divided into six supra‐tentorial regions (left and right anterior, middle and posterior cerebral arteries) and four infra‐tentorial regions (left and right pons/medulla and cerebellum). Infarct volume in each territory was calculated.

The WMH mask was parcellated in regional‐zonal WMH burden by using a bullseye representation (https://github.com/gsanroma/bullseye_pipeline). The white matter and basal ganglia region were divided into a coordinate system composed of 36 distinct parcels, representing a nine‐lobar segmentation with four layers each (Jiménez‐Balado et al. 2022). The interior and external layers represented the most periventricular area and the juxtacortical regions, respectively (see Data S1).

All volumes were normalized by multiplying the volumes by the cohort's mean intracranial volume and dividing by the subject's intracranial volume.

### 2.6 White Matter Tract‐Based Disconnection

To estimate how focal lesions may lead to pathological effects distally from the primary site of lesions, we used a tract‐based structural disconnection approach (Griffis et al. 2021). A publicly available diffusion MRI streamline tractography atlas, constructed using data from 842 Human Connectome project participants, was used (Yeh et al. 2018). Briefly, population‐level streamline trajectories in MNI space were estimated and 550,000 streamline trajectories were obtained by deterministic fiber tracking and classified by a team of neuroanatomists in 66 fiber bundles. The corpus callosum tract was subsequently divided into five segments to enhance the interpretability of callosal disconnection, resulting in a total of 70 tracts (Griffis et al. 2019). For each tract, the number of streamlines passing through the infarct or WMH mask (“disconnected streamlines”) was converted to a percentage of the total number of streamlines assigned to that tract in the tractography atlas. The approach was independently repeated for infarcts and WMH masks, resulting in an estimate of percent disconnection severity for each tract and both masks.

### 2.7 Modeling Latent Disconnection Factors

**FIGURE 1:**
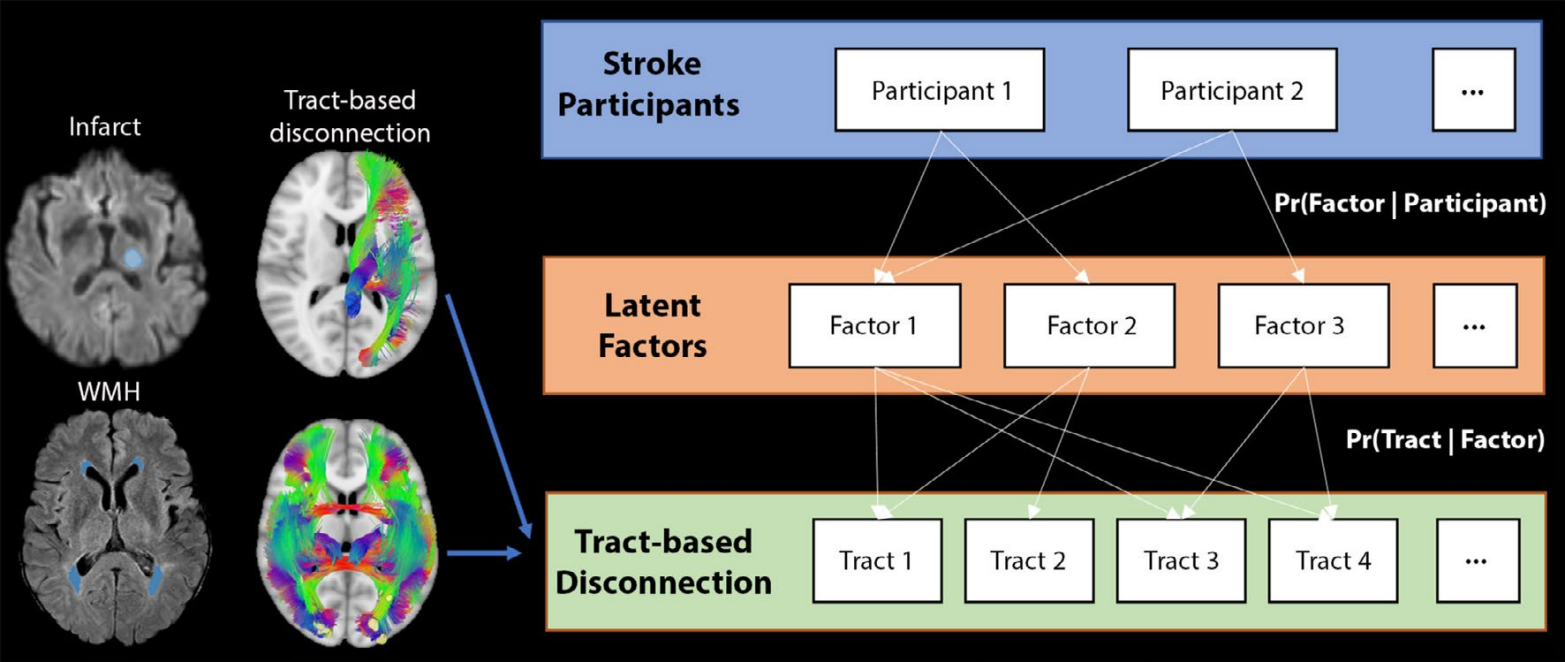
A Bayesian model illustrating participants with stroke, latent factors, and tract‐based disconnection measures. The model assumes that each stroke participant manifests one or more latent factors, and each factor is associated with distinct, albeit possibly overlapping, patterns of tract‐based disconnection induced by infarct (Model 1) or WMH (Model 2).

Since we hypothesized that cognitive impairment in several domains could be linked to a combination of “disconnected” tracts, we used a hierarchical Bayesian model, called LDA, that allows each patient to express one or more latent disconnection factors, each of which is associated with distinct but possibly overlapping tract‐based disconnection patterns (Figure 1) (Zhang et al. 2016). The percentage of disconnected streamlines for each tract was considered as input of the LDA model. Given a user‐defined number of factors *K*, a variational expectation‐maximization algorithm was applied to estimate the probability of an individual expressing a latent factor or factor loading [Pr(Factor | Participant)] and the probability that a factor was associated with disconnection at a tract [Pr(Tract | Factor)]. The final *K* for subsequent analyses was determined by choosing the one that offered the highest stability across runs (Zhang et al. 2016) (Data S1).

### 2.8 Cross‐Sectional Statistical Analysis

All statistical analyses were carried out using the R software (https://www.r‐project.org/; v.4.2.2). As age and educational level differed across groups, they were used as covariates in all statistical analyses and false discovery rate (FDR) was used to correct for multiple comparisons with a statistical significance level set to *p* < 0.05. The following methods were employed to compare the cognitive status across lesion‐based features and to analyze their correlation with cognitive performance.

Logistic regression models were applied to explore how the local and regional volumes and the factor loadings, that is, Pr(Factor | Participant), varied across 6 and 36 months post‐stroke cognitive status. Participants were divided into two groups based on the presence or not of 6‐ and 36‐month PSCI established by the multidisciplinary staff.

To assess the additional value of latent disconnection factors in distinguishing between participants with and without PSCI, two logistic regression models were compared using a likelihood ratio test. In the first model, age, education level, MoCA score, and WMH volume were used as independent variables to predict cognitive status at 6 and 36 months post‐stroke. MoCA score, conducted at 72 h post‐stroke, was included as a continuous variable in the logistic regression model. This was done to control for the initial cognitive status of patients when analyzing the relationship between other variables and the likelihood of developing PSCI. In the second model, significant latent disconnection factors were introduced alongside the independent variables from the first model.

Canonical correlation analysis (CCA) was applied to find an optimal linear combination of the four cognitive domain *z* scores at 6‐ and 36‐month post‐stroke that maximally correlated with lesion‐based features. The statistical significance of CCA was assessed using permutation tests.

### 2.9 Longitudinal Analysis

A longitudinal analysis was conducted to investigate the associative role of white matter and infarct factor loadings in cognitive decline, measured by cognitive status over two time points (6‐ and 36‐month post‐stroke). A logistic mixed‐effects model was used, with cognitive status as the binary outcome variable (0 = no cognitive impairment, 1 = cognitive impairment). The primary predictor was the factor loading, while age and education level were included as covariates to account for potential confounding effects. An interaction term between factor loading and time was included to assess whether the effect of factor loading on cognitive status changed over time. A random intercept for each participant was included to account for within‐subject correlation due to repeated measures. FDR was used to correct for multiple comparisons with a statistical significance level set to *p* < 0.05.

### 2.10 Standard Protocol Approvals, Registrations, and Patient Consents

STROKDEM study was conducted in accordance with the tenets of the Declaration of Helsinki and was approved by local institutional review boards. Prior written informed consent was obtained by all patients or their legal representatives (Comité de Protection des Personnes Nord Ouest IV, Lille, France; reference: 2009‐A00141‐56, March 17, 2009).

### 2.11 Data Availability

The results can be made available on reasonable request and the data have been shared with the STROKOG and METACOHORT consortia (METACOHORTS Consortium 2016; Sachdev et al. 2017).

## 3 Results

### 3.1 Demographic, Clinical, and Imaging Characteristics at Baseline

**FIGURE 2:**
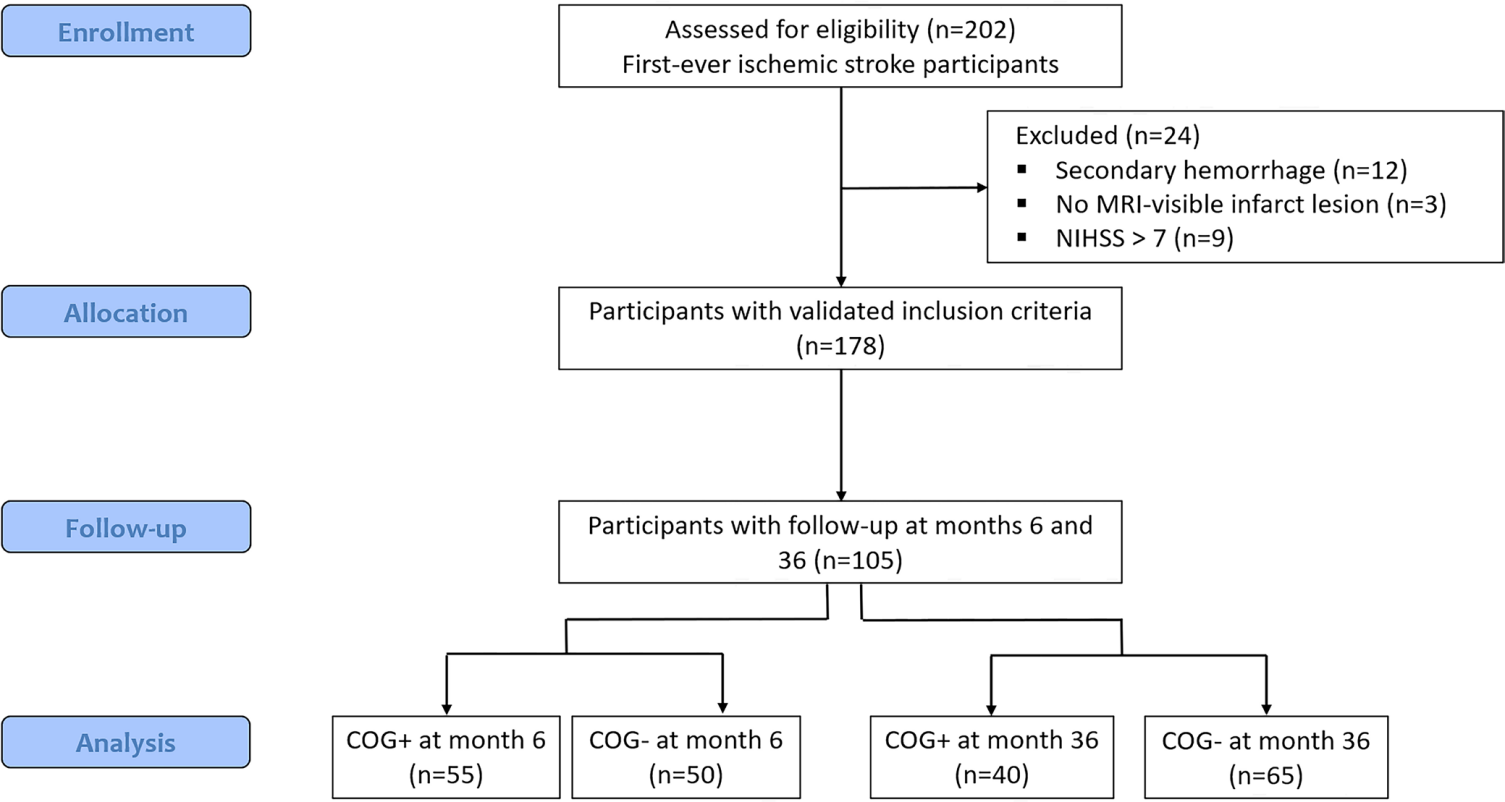
CONSORT flow diagram of the study.

**FIGURE 3:**
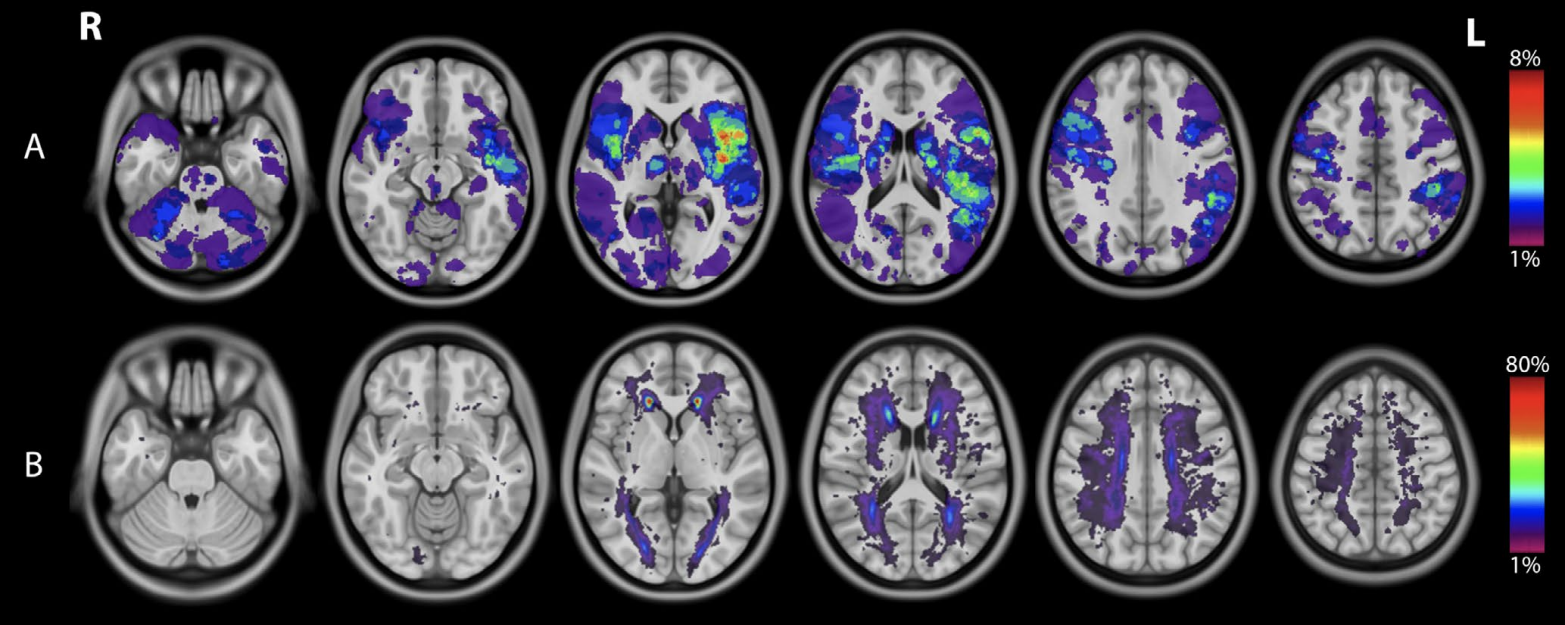
Topography of the infarcts (A) and WMH (B), with a lesion overlay map atlas. Color scale for (A) and (B): number of participants with lesions at each voxel.

Out of the 202 eligible patients, 178 met inclusion criteria, and 105 underwent evaluations at both 6‐ and 36‐month post‐stroke and were included in the current analysis (mean age 63 ± 12; mean educational level 12 years; 31% female) (Figure 2). There were no significant differences between those who did and did not complete the follow‐up evaluations (Table S1). On admission, the study participants had a median NIHSS score of 1 and a mean infarct volume of 10.28 ± 17.10 cm^3^. The infarct was mostly supra‐tentorial (83%) and located in the middle cerebral artery territory (Table S1), but the degree of overlap between infarcts was low (Figure 3A). Participants had a mean WMH volume of 9.01 ± 15.83 cm^3^ with a high degree of overlap between periventricular WMH (Figure 3B). All patients had WMH with a predominance around the lateral ventricles in frontal regions (Figure S1B). No recurrent strokes were observed in our cohort during the 3‐year follow‐up period. This outcome was not a result of selection criteria but likely reflects the effective secondary prevention measures implemented for these patients, as well as the lower risk profile associated with minor ischemic stroke.

### 3.2 Changes in Cognitive Status Between 6‐ and 36‐Month Post‐Stroke

The demographic, clinical, and imaging characteristics at 6‐ and 36‐month post‐stroke are summarized in Table 1.

**TABLE 1:** Demographic, clinical, and cognitive data at 6‐ and 36‐month post‐stroke.

	Month 6			Month 36		
	COG+	COG−	Effect, *p*‐value^a^	COG+	COG−	Effect, *p*‐value^a^
Demographical data						
*n*	55	50		40	65	
Age (years)	65 ± 12	60 ± 11	2.177 0.029	65 ± 11	61 ± 12	1.721 0.085
Males, *n* (%)	36 (65%)	36 (72%)	1.028 0.31	28 (70%)	44 (68%)	0.856 0.35
Educational level (years)	11 ± 4	13 ± 4	−1.972 0.036	10 ± 3	13 ± 4	−2.429 **0.015**
Medical history						
IQCODE score	49 ± 2	48 ± 1	1.037 0.30	49 ± 3	48 ± 2	0.731 0.47
Diabetes mellitus, *n* (%)	9 (16%)	4 (8%)	1.507 0.22	4 (10%)	9 (14%)	0.756 0.38
Hypertension, *n* (%)	34 (62%)	25 (50%)	0.998 0.32	28 (70%)	31 (48%)	1.706 0.19
Body mass index	28 ± 4	27 ± 4	1.060 0.29	27 ± 5	27 ± 3	0.149 0.88
Hyperlipidemia, *n* (%)	25 (45%)	19 (38%)	0.370 0.54	23 (57%)	21 (32%)	3.344 0.067
Tobacco use, *n* (%)	11 (20%)	8 (16%)	0.194 0.66	8 (20%)	11 (17%)	0.001 0.98
Admission data						
NIHSS score	1 (0; 2)	0 (0; 1)	9.037 0.108	0 (0; 1)	1 (0; 2)	8.297 0.141
Fibrinolysis, *n* (%)	17 (31%)	12 (24%)	0.450 0.50	13 (32%)	16 (25%)	0.141 0.71
WMH volume (cm^3^)	9.69 ± 12.08	8.26 ± 19.24	2.056 0.039	13.46 ± 23.00	6.27 ± 8.07	1.991 0.040
Infarct volume (cm^3^)	10.32 ± 18.52	10.24 ± 15.56	0.867 0.39	12.64 ± 21.83	8.83 ± 13.34	0.708 0.48
Left hemisphere stroke, *n* (%)	31 (56%)	25 (50%)	0.700 0.40	20 (50%)	36 (55%)	0.045 0.83
Supra‐tentorial stroke, *n* (%)	46 (84%)	41 (82%)	0.100 0.76	33 (83%)	54 (83%)	0.081 0.78
Affected vascular territories, *n*			7.486 0.587			7.280 0.61
Left MCA	23	15		16	22	
Right MCA	13	14		10	17	
Left PCA	4	3		2	5	
Right PCA	5	3		4	4	
Left ACA	1	2		1	2	
Right ACA	0	4		0	4	
Left cerebellum	2	3		3	2	
Right cerebellum	3	4		1	6	
Left pons	2	1		2	1	
Right pons	2	1		1	2	
Cognitive domains (*z* scores)						
Memory	−0.42 ± 0.92	0.49 ± 0.63	−2.45 **3.22** ^**−6**^	−0.57 ± 1.04	0.48 ± 0.72	−4.66 **3.22** ^**−6**^
Attention/executive functions	−1.14 ± 1.57	−0.02 ± 0.70	−3.54 **4.00** ^**−4**^	−1.05 ± 1.66	0.09 ± 0.72	−4.44 **8.82** ^**−6**^
Visuospatial functions	−0.04 ± 0.78	0.33 ± 0.44	−3.24 **0.001**	−0.18 ± 0.92	0.42 ± 0.38	−3.98 **6.96** ^**−5**^
Language	−0.54 ± 0.90	0.14 ± 0.56	−4.31 **1.62** ^**−5**^	−0.46 ± 0.69	0.15 ± 0.57	−4.10 **4.05** ^**−5**^

*Note:* Quantitative variables are quoted as the mean ± standard deviation or the median (interquartile range). Uncorrected *p* values were displayed and *p* values < 0.05 corrected for multiple comparisons are given in bold type.

Abbreviations: ACA: anterior cerebral artery; COG+: participants with cognitive impairment at 6 or 36 months post‐stroke; COG−: participants without cognitive impairment at 6 or 36 months post‐stroke; IQCODE: Informant Questionnaire on Cognitive Decline in the Elderly; MCA: middle cerebral artery; NIHSS: National Institute of Health Stroke Scale; PCA: posterior cerebral artery; WMH: white matter hyperintensity.

^a^ A *χ* ^2^ test and Wilcoxon rank sum test were applied to categorical and quantitative variables, respectively. The effect corresponded to the estimated effect size of each variable on the outcome of interest using *χ* ^2^ or Wilcoxon statistics.

Six months after stroke, 55 participants (52%) had PSCI. They were older (*p* = 0.038) and had a lower educational level (*p* = 0.036) than participants without PSCI.

Thirty‐six months after stroke, 40 participants (38%) had PSCI with a lower educational level (*p* = 0.002) and were slightly older (*p* = 0.064) than participants without PSCI. As expected, participants with PSCI showed lower *z* scores for all cognitive domains than participants without PSCI at 6‐ and 36‐month post‐stroke (*p* < 0.008 and *p* < 4 × 10^−5^, respectively). In the following results, age and educational level were used as covariates in statistical analyses.

Regarding the temporal dynamics of cognitive impairment between 6‐ and 36‐month post‐stroke, 35 participants were converters, with 10 developing new‐onset cognitive impairment at 36 months and 25 having cognitive impairment at 6 months but not at 36 months. Seventy participants were non‐converters, with 40 showing no cognitive impairment at either time points and 30 experiencing cognitive impairment at both time points.

### 3.3 Associations Between Lesion Volume/Location and Cognitive Impairment at 6‐ and 36‐Month Post‐Stroke

Infarct volume and territory showed no association with 6‐ and 36‐month PSCI (Table 2 and Figure S1A). While participants with PSCI at both time points exhibited higher WMH volumes (*p* = 0.041 and *p* = 0.021, Table 1), this association did not remain significant after correcting for age and educational level (Table 2). No significant association was observed between WMH loads in brain parcels and PSCI (Figure S1B).

**TABLE 2:** Logistic regression results for cognitive status at 6‐ and 36‐month post‐stroke.

	Month 6			Month 36		
Independent variable	*β* ± SE	OR [95% CI]	*p* value	*β* ± SE	OR [95% CI]	*p* value
Model for infarct volume						
Infarct volume	0.129 ± 0.106	1.14 [0.92; 1.41]	0.30	0.095 ± 0.107	1.10 [0.89; 1.37]	0.51
Age	0.037 ± 0.019	1.04 [1.00; 1.08]	0.09	0.028 ± 0.019	1.03 [0.99; 1.07]	0.26
Educational level	−0.104 ± 0.053	0.90 [0.81; 1.00]	0.09	−0.122 ± 0.057	0.88 [0.78; 0.98]	0.13
Model for WMH volume						
WMH volume	0.209 ± 0.173	1.23 [0.88; 1.75]	0.45	0.264 ± 0.180	1.30 [0.93; 1.89]	0.28
Age	0.017 ± 0.023	1.02 [0.97; 1.07]	0.61	0.005 ± 0.023	1.00 [0.96; 1.05]	0.85
Educational level	−0.097 ± 0.052	0.91 [0.81; 1.00]	0.25	−0.118 ± 0.058	0.89 [0.79; 0.99]	0.16
Model for tract‐based disconnection by infarct (factor loading)						
Factor 1	−0.602 ± 0.453	0.55 [0.22; 1.32]	0.24	0.139 ± 0.455	1.15 [0.47; 2.82]	0.76
Age	0.036 ± 0.018	1.04 [1.00; 1.08]	0.16	0.026 ± 0.019	1.03 [0.99; 1.07]	0.32
Educational level	−0.092 ± 0.053	0.91 [0.82; 1.01]	0.16	−0.120 ± 0.057	0.89 [0.79; 0.98]	0.13
Factor 2	0.787 ± 0.443	2.20 [0.93; 5.33]	0.11	0.131 ± 0.438	1.14 [0.48; 2.70]	0.76
Age	0.033 ± 0.018	1.03 [1.00; 1.07]	0.11	0.026 ± 0.019	1.03 [0.99; 1.07]	0.34
Educational level	−0.097 ± 0.053	0.91 [0.81; 1.00]	0.10	−0.118 ± 0.057	0.89 [0.79; 0.99]	0.15
Factor 3	−0.439 ± 0.623	0.64 [0.18; 2.19]	0.64	−0.547 ± 0.648	0.58 [0.15; 2.00]	0.53
Age	0.033 ± 0.018	1.03 [1.00; 1.07]	0.15	0.024 ± 0.019	1.02 [0.99; 1.06]	0.40
Educational level	−0.103 ± 0.052	0.90 [0.81; 1.00]	0.15	−0.123 ± 0.057	0.88 [0.78; 0.98]	0.12
Model for tract‐based disconnection by WMH (factor loading)						
Factor 1	−1.451 ± 0.664	0.23 [0.06; 0.84]	0.12	−2.696 ± 0.770	0.07 [0.01; 0.28]	**0.002**
Age	0.022 ± 0.019	1.02 [0.98; 1.06]	0.33	0.004 ± 0.020	1.00 [0.96; 1.05]	0.83
Educational level	−0.095 ± 0.053	0.91 [0.82; 1.01]	0.15	−0.121 ± 0.060	0.89 [0.78; 0.99]	0.09
Factor 2	2.300 ± 0.802	9.96 [2.21; 52.4]	**0.02**	2.507 ± 0.794	12.27 [2.8; 63.4]	**0.006**
Age	0.033 ± 0.019	1.03 [1.00; 1.07]	0.16	0.026 ± 0.019	1.03 [0.99; 1.07]	0.249
Educational level	−0.082 ± 0.055	0.92 [0.82; 1.03]	0.18	−0.103 ± 0.059	0.90 [0.80; 1.01]	0.16
Factor 3	−0.642 ± 0.941	0.53 [0.08; 3.29]	0.50	0.924 ± 0.947	2.52 [0.39; 16.7]	0.45
Age	0.040 ± 0.020	1.04 [1.00; 1.08]	0.12	0.019 ± 0.020	1.02 [0.98; 1.06]	0.45
Educational level	−0.097 ± 0.052	0.91 [0.82; 1.00]	0.12	−0.124 ± 0.057	0.88 [0.78; 0.98]	0.12

*Note:* Logistic regression model: Cog ~ Independent variable + Age + Edu. *p* values < 0.05 corrected by FDR are given in bold type.

Abbreviations: *β*: estimated coefficients for each independent variable, indicating the direction and strength of the relationship with the cognitive status; CI: confidence interval; Cog: cognitive status (0 for no PSCI and 1 for PSCI); Edu: educational level (in years); OR: odds ratio; SE, standard error of the coefficient *β*.

### 3.4 Latent Disconnection Factors and Associations With Cognitive Impairment at 6‐ and 36‐Month Post‐Stroke

The Bayesian model was applied to infarcts and WMH disconnected tracts with varying numbers of latent factors (*K* = 2–10). In both Bayesian models, the 3‐factor model demonstrated the highest stability.

**FIGURE 4:**
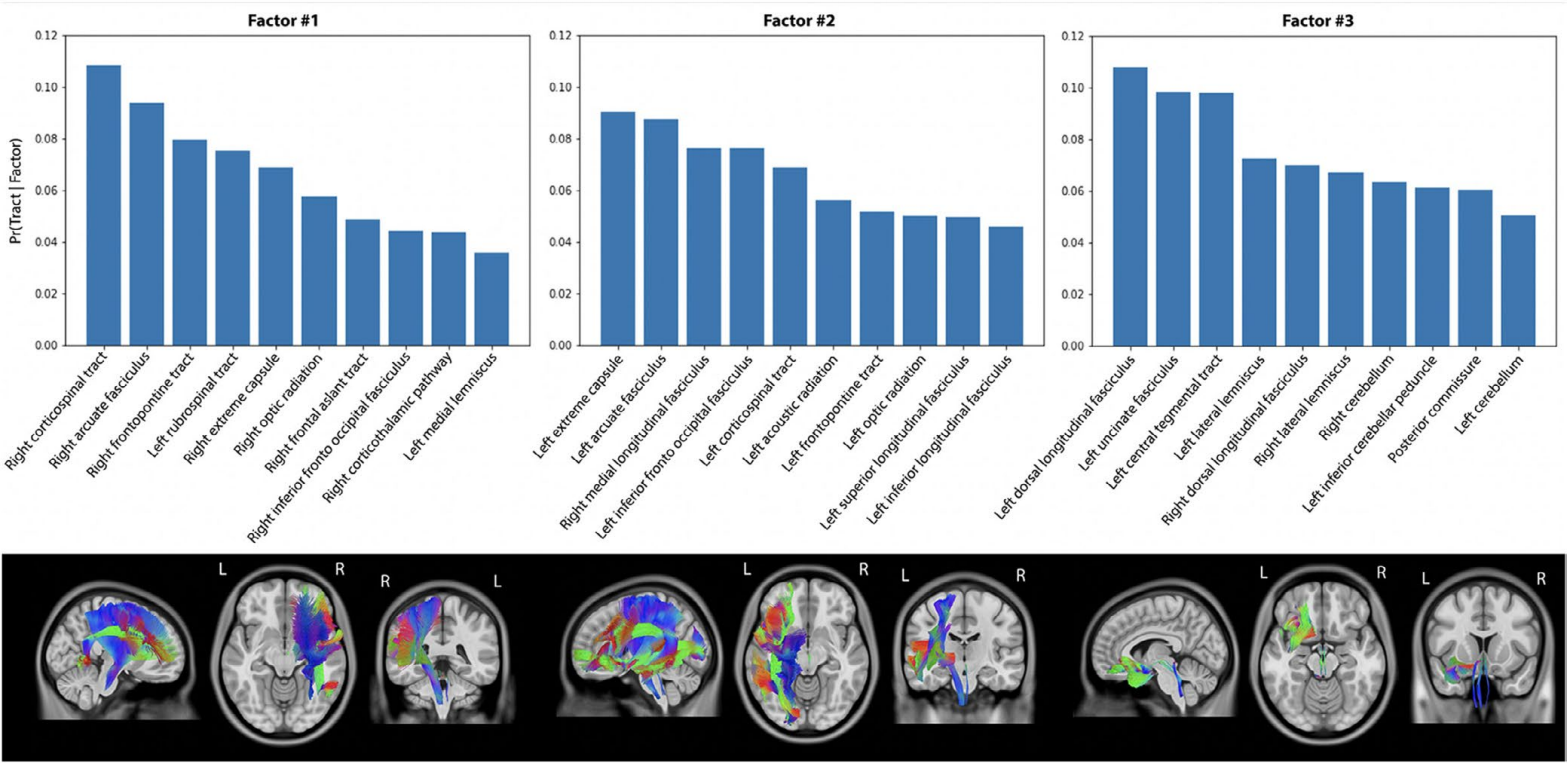
The three latent tract‐based disconnection factor estimated from infarcts of participants. The first line displays the probability of disconnection at that tract for a particular factor [i.e., Pr(Tract | Factor)]. The second line displays the five tracts with the highest probability of disconnection.

The Bayesian model identified three latent factors using disconnected tracts by infarct that differed with respect to hemisphere and infra‐tentorial signatures (Figure 4). Factor 1 was defined almost exclusively by disconnections of right hemisphere tracts. The 10 most severe disconnected tracts accounted for 66% of the disconnection load (i.e., the summation of posterior probability across tracts), defined by 4 projection, 4 association and 2 brainstem tracts. Factor 2 was mostly the symmetric of Factor 1 for the left hemisphere. The 10 most severely disconnected tracts accounted for 65% of the disconnection load, defined by 4 projection and 6 association tracts. Factor 3 was mainly defined by disconnections of infra‐tentorial tracts. The 10 most severe disconnected tracts accounted for 75% of the disconnection load, defined by 3 association, 3 brainstem, 3 cerebellum, and 1 commissural tracts. Factor loadings were similar between cognitive status groups at 6‐ and 36‐month post‐stroke (Table 2).

**FIGURE 5:**
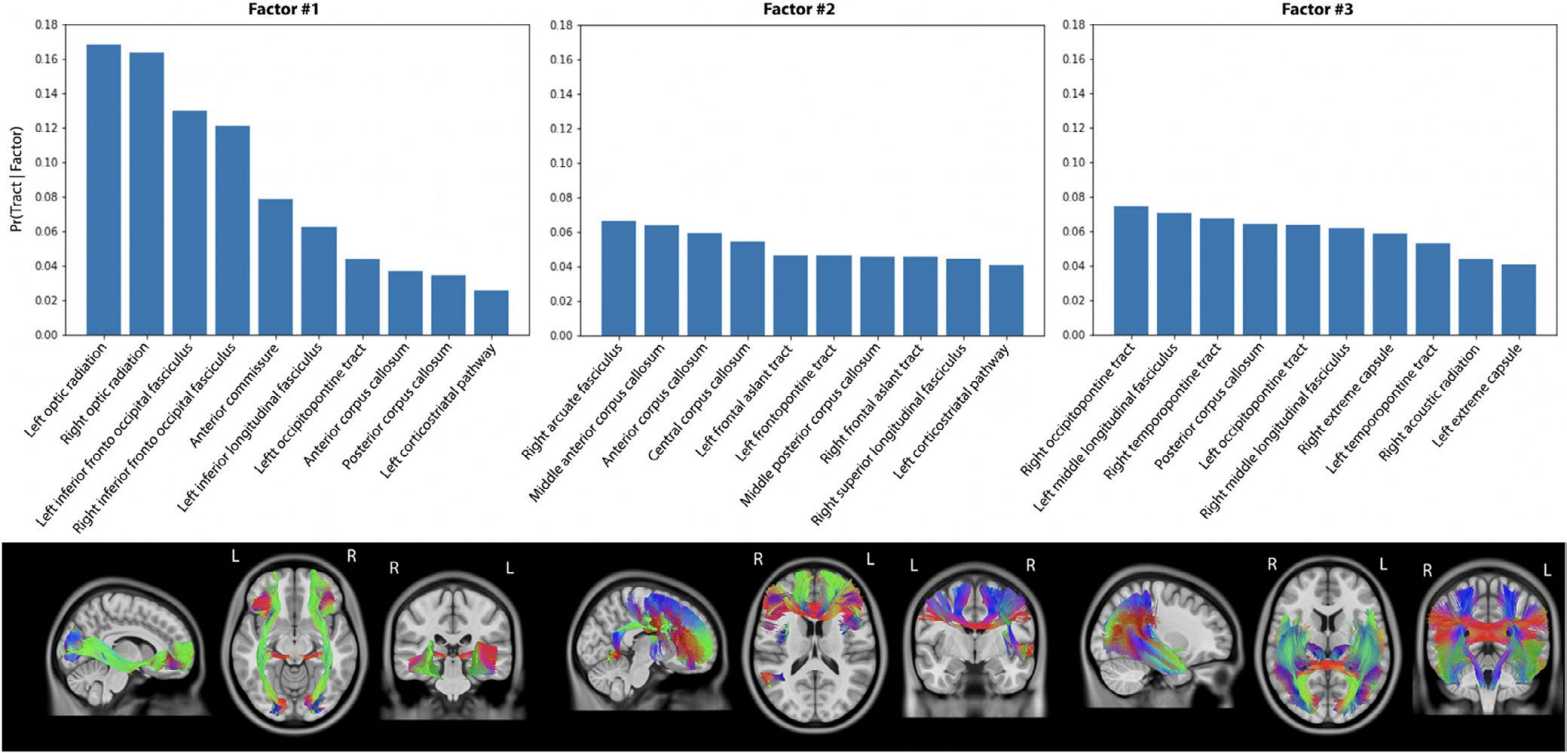
The three latent tract‐based disconnection factor estimated from WMH of participants. The first line displays the probability of disconnection at that tract for a particular factor [i.e., Pr(Tract | Factor)]. The second line displays the five tracts with the highest probability of disconnection.

Using disconnected tracts affected by WMH, the Bayesian model identified three latent factors distinguished by commissural and disconnection load (Figure 5). For Factor 1, over half of the disconnection load was attributed to two tracts, namely the optic radiation and inferior fronto‐occipital fasciculus. Factor 2 was predominantly characterized by disconnections in commissural and frontal tracts, with the 10 most severe disconnected tracts accounting for 51% of the load—comprising 4 commissural, 4 association, and 2 projection tracts. Factor 3 was mainly defined by disconnections in temporo‐occipital tracts with the 10 most severe disconnected tracts accounting for 60% of the load—comprising 5 projection, 4 association, and 1 commissural tracts. Factor 2 loading was higher in participants with 6‐ and 36‐month PSCI (Table 2), while Factor 1 loading was lower specifically for participants with 36‐month PSCI.

### 3.5 Added Value of Latent Disconnection Factors for PSCI Explainability Compared to Other Demographic and Clinical Variables

The model using Factor 2 derived from WMH‐induced disconnection outperformed the model using only demographic and clinical variables in predicting of 6‐ and 36‐month PSCI (Table 3). At 6 months, Factor 2 was the only significant predictor of PSCI. However, at 36 months, both MOCA and Factor 2 emerged as significant predictors of PSCI.

**TABLE 3:** Likelihood ratio test results for comparison between logistic regression models.

Model	Log‐likelihood	*χ* ^2^	*p* value
Month 6			
Model 1: Cog ~ MOCA + WMH + Factor 2 + Age + Edu *p*_MOCA_ = 0.053; *p*_WMH_ = 0.576; ***p***_**Factor 2**_ **= 0.013**; *p*_Age_ = 0.328; *p*_Edu_ = 0.224^a^	−60.655	6.79	**0.0092**
Model 2: Cog ~ MOCA + WMH + Age + Edu ***p***_**MOCA**_ **= 0.031**; *p*_WMH_ = 0.363; *p*_Age_ = 0.358; *p*_Edu_ = 0.132^a^	−64.047		
Month 36			
Model 1: Cog ~ MOCA + WMH + Factor 2 + Age + Edu ***p***_**MOCA**_ **= 0.017**; *p*_WMH_ = 0.396; ***p***_**Factor 2**_ **= 0.006**; *p*_Age_ = 0.618; *p*_Edu_ = 0.150^a^	−56.829	8.30	**0.0039**
Model 2: Cog ~ MOCA + WMH + Age + Edu ***p***_**MOCA**_ **= 0.010**; *p*_WMH_ = 0.251; *p*_Age_ = 0.650; *p*_Edu_ = 0.094^a^	−60.977		

*Note:* We assumed that the likelihood ratio statistic follows a *χ* ^2^ distribution. *p* value < 0.05 means that Model 1 is significantly better than Model 2, suggesting that including Factor 2 from tract‐based disconnection by WMH as an independent variable significantly improves the model's fit for predicting cognitive status. *p* values < 0.05 corrected by FDR are given in bold type.

Abbreviations: Factor 2: Factor 2 from tract‐based disconnection by WMH; Edu: educational level; WMH: white matter hyperintensity volume.

^a^ *p* values of logistic regression model.

In Table 2, both Factor 1 and Factor 2 were significantly associated with PSCI at 36 months. However, only Factor 2 was included in Model 1 due to the high correlation between Factor 1 and Factor 2 (*R* = −0.71) and for interpretability. The association between Factor 2 and PSCI is positive, unlike Factor 1, meaning that subjects with a high load of Factor 2 have a higher probability of having PSCI. We performed a sensitivity analysis that included both Factor 1 and Factor 2 in the likelihood ratio test model at 36 months. The results indicated that including Factor 1 did not significantly improve the model's predicitive power (Table S2).

### 3.6 Associations Between Significant Markers of PSCI and Cognitive Domains at 6‐ and 36‐Month Post‐Stroke

**FIGURE 6:**
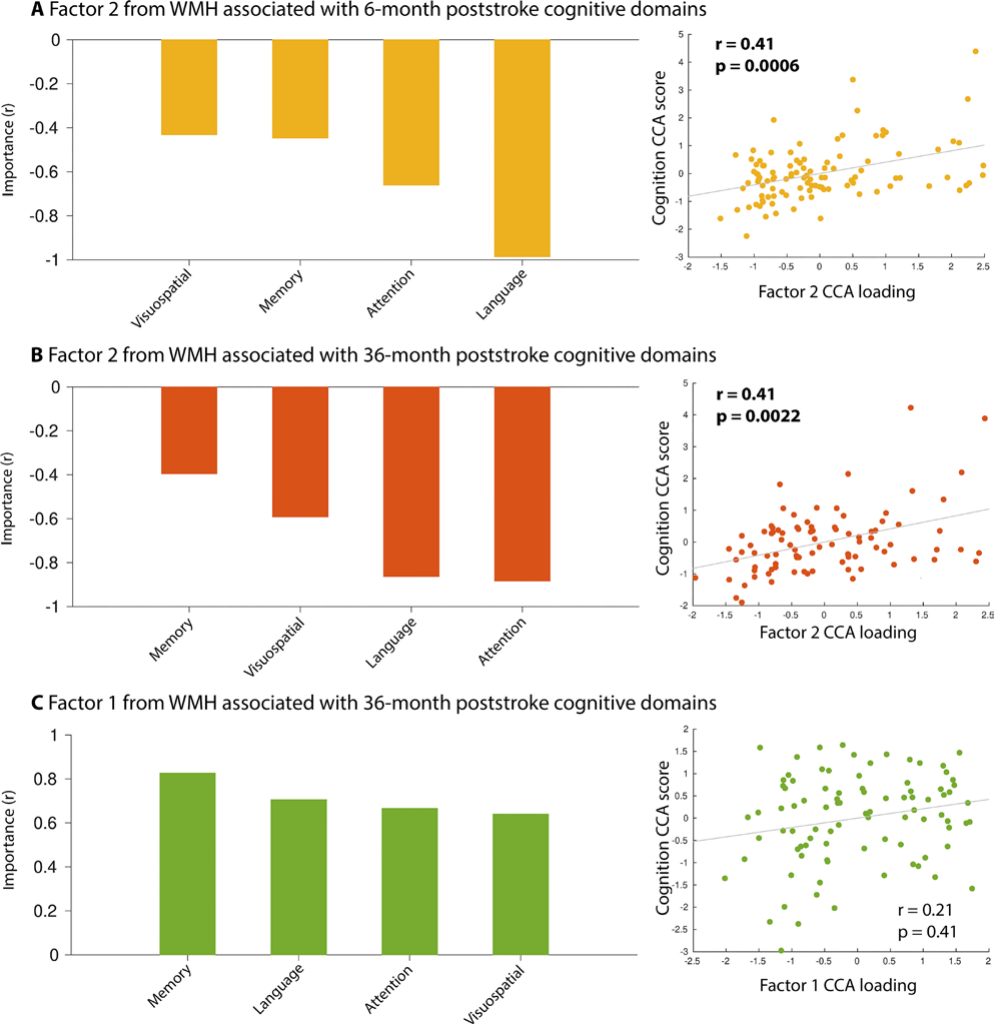
Canonical correlation analyses between the four cognitive domain *z* scores and the significant lesion‐based features from the comparisons of 6 and 36 months PSCI. We display the results between the loadings of factors identified from disconnection by WMH and four groups of cognitive *z* score. Positive correlation suggests that a higher loading was associated with greater impairment. The scatter plots show the relationships between CCA cognitive score and CCA loading, where each dot represents a participant. For example, Factor 2 was associated with worse attention, language, and visuospatial functions at 36 months post‐stroke (B).

To explore associations between previously identified significant markers and cognitive domains, CCA was conducted between each marker and the *z* scores specific to four cognitive domains. Factor 2 derived from WMH‐induced disconnection was linked to more pronounced impairment in language and attention domains at 6 months post‐stroke (Figure 6A) and to greater impairment in attention, language, and visuospatial domains at 36 months post‐stroke (Figure 6B). Factor 1 loading did not exhibit any association with cognitive domains (Figure 6C).

### 3.7 Associative Effect of Neuroimaging Markers on the Change in Cognitive Status Between 6‐ and 36‐Month Post‐Stroke

Cognitive status changed in 35 participants (33%) between 6 and 36 months post‐stroke (Table S3). Of these, 25 participants who had cognitive impairment at 6 months no longer exhibited impairment at 36 months, while 10 participants without cognitive impairment at 6 months developed impairment by 36 months. Among the non‐converters, 30 participants (29%) exhibited cognitive impairment at both 6‐ and 36‐month post‐stroke. Factor 2 derived from WMH‐induced disconnection was significantly associated with higher odds of cognitive impairment (odds ratio = 18.6, corrected *p* value = 0.03) (Table 4). Age and education level, included as covariates, showed nonsignificant trends toward higher and lower odds of cognitive impairment, respectively. The interaction between factor loading and time did not demonstrate a significant change in the impact of WMH‐induced disconnection on cognitive impairment over time.

**TABLE 4:** Longitudinal analysis of cognitive status changes associated with Factor 2 derived from WMH‐induced disconnection.

Models: Cog ~ Factor 2 × Time + Age + Edu + 1 | Participants	*t*‐value	Odds ratio	*p* value
Intercept	−1.17	0.19	0.29
Factor 2	2.72	18.60	**0.03**
Time	−1.47	0.46	0.21
Factor 2 × Time	0.20	1.29	0.84
Age	1.96	1.04	0.10
Educational level	−2.06	0.89	0.10

*Note: p* values < 0.05 corrected by FDR are given in bold type.

Abbreviations: Cog: Cognitive status at 6‐ and 36‐month post‐stroke (0: without cognitive impairment, 1: with cognitive impairment); Edu: educational level; Factor 2: Factor 2 from tract‐based disconnection by WMH.

## 4 Discussion

In this study, while we did not find a significant relationship between infarct‐based features and 6‐ or 36‐month PSCI using the current method, we identified a disconnection factor induced by WMH in the commissural and frontal tracts, as well as the right superior longitudinal fasciculus, that was significantly associated with PSCI at both time points, particularly in the executive/attention, language, and visuospatial domains. These findings suggest that in minor strokes, ongoing chronic cerebrovascular lesions present at stroke onset may play a more critical role in long‐term PSCI than the acute infarct characteristics themselves.

Patients with minor stroke have higher risk of cognitive impairment and even dementia than the general population (Leng and Wang 2023; Pendlebury et al. 2019). Understanding the underlying mechanisms of cognitive impairments following a minor stroke is challenging due to the size of the stroke. Indeed, infarct volume and location are common markers of PSCI (Weaver et al. 2021). However, the effects are more pronounced for severe strokes than for minor strokes (Pendlebury et al. 2019). Additionally, the approach used, which relies on voxel‐based lesion‐symptom mapping, requires a sufficient brain overlap of infarcts to perform statistical comparisons. Given the small sizes of infarcts in our population and the limited number of subjects, there was a low overlap between lesions. Therefore, we resorted to alternative approaches.

In this study, we developed a disconnection‐symptom mapping technique to address the relatively small sample size and to identify association with remote white matter bundles where structural disconnection is likely to occur. One strength of this approach is the requirement of only conventional MR sequences. Given the heterogeneity in the rate of progression of PSCI and the involvement of multiple cognitive domains (Leng and Wang 2023; Lopes et al. 2021), we used a data‐driven Bayesian framework, called LDA, to model the possibility that multiple latent factors are expressed to varying degrees within a subject.

As expected, the infarct volume and location were not associated with PSCI in our population. While the disconnection approach allowed us to statistically assess the potential impact of infarcts on PSCI, the lack of association limits our ability to draw definitive conclusions about the effects of infarcts in patients with minor strokes. This lack of association may be partly due to the relatively low overlap of infarct locations in our cohort, which also reduced the effectiveness of the Bayesian model based on infarct‐induced white matter tract disconnection. The three‐factor model identified distinct disconnection topographies corresponding to the damaged vascular territory, with two factors reflecting symmetric disconnections in left or right supra‐tentorial regions and the third factor representing infra‐tentorial strokes. Consequently, the assumption within the Bayesian model that patients could exhibit multiple disconnection factors was constrained, potentially diminishing its explanatory power in this context. Given that the three infarct‐based latent factors align with the topography of the infarct locations, we tested the association between PSCI status and these infarct‐based latent factors, including in each logistic regression model only the patients who were susceptible to express the factor (see Data S1). However, the association remained nonsignificant (Table S4).

In our study, PSCI was associated with WMH‐based features. WMH load is considered as a correlate of long‐term cognitive impairment (Sachdev et al. 2007; Schmidt et al. 2003). However, we did not find any association between total WMH volume and 6‐ or 36‐month PSCI when adjusted for age and education level. A previous study on participants with minor stroke showed a relationship between the total WMH load and persistent cognitive impairment (> 30 days), but WMH volume no longer associated with PSCI when adjusted for age (Sivakumar et al. 2017). Older age and lower levels of education are consistent risk factors for PSCI (Gottesman and Hillis 2010). Our results were consistent with this statement as participants with 6‐month PSCI had higher WMH volume than those without 6‐month PSCI when adjusted only for education level (*p* = 0.035).

Tract‐based disconnection induced by WMH was the marker most strongly associated with long‐term PSCI, supporting the assumption that WMH affect cortical connectivity by diminishing efficiency of neural transmission, resulting in cognitive impairment (Black, Gao, and Bilbao 2009). One latent factor (Factor 2) composed of commissural, frontal tracts, and right superior longitudinal fasciculus was associated with cognitive domains at 6 and 36 months after stroke, and more specifically with attention and language domains 6‐month post‐stroke and with visuospatial, language, and attention 36‐month post‐stroke. Our results are consistent with a recent study using the same dataset showing a relationship between functional connectivity from frontal regions at 6 months post‐stroke and cognitive *z* scores of attention and visuospatial domains at 36 months post‐stroke (Lopes et al. 2021). Vascular lesions within corpus callosum have been associated with worse performance on cognition and reduced microstructural white matter integrity within both the corpus callosum and the whole‐brain white matter (Freeze et al. 2022). Moreover, Zhao et al. (2018) showed that in long‐term PSCI WMH were mainly located in the corpus callosum. The integrity of commissural pathways are essential for selective attention as well as processing and integration of visuospatial information (Schulte and Müller‐Oehring 2010). Language impairments at 6‐ and 36‐month post‐stroke were also highly associated with the Factor 2 loading. The arcuate fasciculus and the frontal aslant tract were among the 10 most severe disconnected tracts represented by Factor 2. The arcuate fasciculus has a major implication in language processing (Catani and Mesulam 2008) by connecting the key speech production region in the frontal lobe with the speech comprehension region in the posterior temporal lobe (Geschwind 1970). The frontal aslant tract connects the inferior frontal gyrus with the superior frontal gyrus and cingulate gyrus and sulcus. This tract has been found to be highly associated with speech initiation, verbal fluency, stuttering, and executive functions (Dick et al. 2019). The right superior longitudinal fasciculus is a major white matter pathway comprising three branches (Wang et al. 2016), each contributing to different aspects of cognitive function, particularly attention and visuospatial processing (Martín‐Signes, Paz‐Alonso, and Chica 2019; Parlatini et al. 2017). Disconnection of this tract can disrupt the pathways, leading to deficits in attention and visuospatial functions, which are crucial components of cognitive function. To further explore the role of the superior longitudinal fasciculus, future studies could employ advanced neuroimaging techniques to examine the specific contributions of each branch to PSCI.

Our study includes limitations. Although our tract‐based structural disconnection approach is popular and validated, it is an indirect method to assess the degree of disconnection of each tract by assuming that all brains have the same white matter anatomy. Atlas‐based approaches are not sensitive against potential biases arising from interindividual differences. Indeed, it is important to highlight that the white matter tract atlas used in our study was based on very high‐quality data with 90 direction high‐angular resolution diffusion imaging, and it was derived from a large sample of participants (*N* = 842 participants). While the atlas provides valuable information for our current analysis, a further study could explore the potential benefits of creating a white matter tract atlas specifically tailored to the demographics of the patient population under investigation (Taghvaei et al. 2023). Furthermore, tract‐based structural disconnection approach assumes an axonal damage, while WMH are heterogenous lesions associated with structural brain changes such as gliosis, demyelination, and vacuolation, in addition to axonal degeneration (Gouw et al. 2011; Prins and Scheltens 2015). In a supplemental analysis, we have analyzed the correlation between WMH and underlying axonal integrity in our study using diffusion tensor imaging (DTI) data, specifically the measure of fractional anisotropy (FA), the AD, and the RD. Median voxel values for DTI metrics were extracted from contralesional NAWM and WMH, as well as ipsilesional NAWM and infarct. The results show that AD and RD were significantly increased in WMH compared to NAWM, while FA was significantly decreased in WMH compared to NAWM (Table S5). These results are in agreement with the study by Etherton et al. (2021), which reported similar differences in a cohort of 319 stroke patients. We found the same differences in infarct compared to NAWM (Table S5). Secondly, the accuracy of tract anatomy may be affected by the relatively large voxel size of the DWI sequence (14.4 mm^3^) compared to the FLAIR sequence (2.5 mm^3^). Moreover, while the use of routine clinical MRI data is a strength of our study, it is important to acknowledge that MRI is not universally available or routinely performed in all hospitals following a stroke. This variability may limit the generalizability of our findings and the clinical applicability of our models in settings where MRI is not part of standard stroke care. Thirdly, the cross‐sectional and longitudinal analyses highlighted the importance of Factor 2, derived from WMH‐induced disconnection, as a key determinant of PSCI. The nonsignifcant interaction between factor loading and time point suggests that the influence of this factor on cognitive status remained consistent over time. However, these findings underscore the need for further investigation into potential modifiers, such as age and education. Finally, the hodological (tract‐focused) approach used in our study assumes that infarct or WMH syndromes may extend into axonal pathways connecting distant brain regions. While this approach is not novel, its utility still requires validation in large cohorts. Nevertheless, in our study, the hodological approach proved more relevant than the topological (region‐based) approach for understanding the relationship between structural disconnection and cognitive impairment (Dick et al. 2019). Furthermore, even when there is an overlap of information, tract‐based disconnection measures remain valuable from a neuroscience perspective, especially in investigating alterations in brain networks.

Future studies could benefit from linking WMH‐induced disconnection markers to secondary degeneration processes assessed through advanced imaging sequences, such as brain atrophy on 3D T1W images (Aribisala et al. 2013), iron load on quantitative susceptibility images (Kuchcinski et al. 2017), functional connectivity on resting‐state fMRI (Bournonville et al. 2018), and structural connectivity on DWI (Zhao et al. 2023).

## 5 Conclusion

The present study identified a marker of tract‐based disconnection information induced by WMH and associated with long‐term PSCI in patients with minor strokes. Further studies on larger cohorts of patients are needed to confirm these findings. This marker, based on conventional MR sequences, has the potential to be integrated into clinical practice. With further development, it could aid in identifying patients at high risk of long‐term cognitive decline after stroke. Such patients may benefit from early cognitive rehabilitation and personalized therapy.

## Supporting information


**Data S1.** Supporting Information.

## Data Availability

The data that support the findings of this study are available on request from the corresponding author. The data are not publicly available due to privacy or ethical restrictions.
