# Supplementary material for: Long‐Term Post‐Stroke Cognition in Patients With Minor Ischemic Stroke is Related to Tract‐Based Disconnection Induced by White Matter Hyperintensities
Source: Hum Brain Mapp. 2025 Jan 27;46(2):e70138. doi: 10.1002/hbm.70138 (PMC11770330; doi:10.1002/hbm.70138)
Supplement: Supplementary file 1 — Data S1. Supporting Information. [file HBM-46-e70138-s001.docx]

**Supplemental Material**

Supplemental methods

Neuropsychological Assessment

Infarcts and WMH Location

Modelling latent disconnection factors

Selection of the number of latent factors

Supplemental results

Table S1. Baseline characteristics of members of the Study of Factors Influencing Poststroke Dementia (STROKDEM) cohort excluded from or included in the present study

Table S2. Likelihood ratio test results for comparison between logistic regression models

Table S3. Demographic and clinical data at 36 months poststroke for converter and non-converter participants.

Table S4. Associations between infarct-based latent factors and cognitive PSCI status at 6- and 36-month poststroke using subgroups of patients

Table S5. Diffusivity anistropy metrics in normal-appearing white matter, WMH, and infarct

References

**Supplemental methods**

## Neuropsychological Assessment

The participants’ cognitive functions, including executive functions/attention, memory, language, and visuospatial abilities, were assessed 6- and 36-month post-stroke using a battery of neuropsychological tests (T.D.). The executive function/attention tests were the Trail-Making Test parts A and B, a version of the Stroop test, and the “code” subtest from Wechsler Adult Intelligence Scale III^1,2^. For the memory domain, the total of the three free recall trials, the delayed free recall from the Free and Cued Selective Reminding Test, and the delayed recall score from the Rey-Osterrieth complex figure test were recorded^3,4^. The language domain score was built with scores from the DO 80, semantic fluency (animal) and phonemic fluency (p words) tests^5,6^. The incomplete letter and number location subtests from the Visual Object and Space Perception and the Rey-Osterrieth complex figure test were used to assess visuospatial abilities^7^.

For each participant with complete data, test-specific z scores based on available norms adjusted for age, sex, and education were calculated and averaged by domain to obtain summary domain-specific z scores. Each participant’s cognitive status was reviewed in a multidisciplinary staff meeting at 6- and 36-month post-stroke, based on any complaints and symptoms during clinical examination, and the neuropsychological test results.

## Infarcts and WMH Location

A vascular territory template in MNI space was used to create an incidence map of infarct location^8^. The template was divided into 6 supratentorial regions (left and right anterior, middle and posterior cerebral arteries) and 4 infratentorial regions (left and right pons/medulla and cerebellum).

The WMH mask was parcellated in regional-zonal WMH burden by using a bullseye representation (https://github.com/gsanroma/bullseye_pipeline). The white matter and basal ganglia region were divided into a coordinate system composed of 36 distinct parcels, representing a nine-lobar segmentation with four layers each^9^. Frontal, parietal, temporal and occipital regions of white matter were obtained with FreeSurfer’s pipeline, as well as basal ganglia region labeled according to caudate, putamen, thalamus and globus pallidus subcortical segmentations. The concentric rings in the bullseye plot were defined by dividing the area between the ventricular surface and the cortical sheet into four equidistant layers. The interior and external layers represented the most periventricular area and the juxtacortical regions, respectively.

All volumes were normalized by multiplying the volumes by the mean population’s intracranial volume and dividing by the subject’s intracranial volume.

## Modelling latent disconnection factors

Since we hypothesized that cognitive impairment in several domains could be linked to a combination of « disconnected » tracts, we used a hierarchical Bayesian model, called Latent Dirichlet Allocation (LDA), that allows each patient to express one or more latent disconnection factors, each of which is associated with distinct but possibly overlapping tract-based disconnection patterns^10^. We applied the LDA model originally developed for identifying topics in a collection of documents^11^, and extended to neuroimaging in a recent study^10^. The percentage of disconnected streamlines for each tract was considered as input of the LDA model. Given a user-defined number of factors K, a variational expectation-maximization algorithm was applied to estimate the probability of an individual expressing a latent factor or factor loading [Pr(Factor │ Participant)] and the probability that a factor was associated with disconnection at a tract [Pr(Tract │ Factor)]. A range of latent factors K from 1 to 4 was evaluated and, for each K, the LDA model was rerun with 100 different random initializations. The solution closest to the remaining 99 runs based on product-moment correlation of the factors was selected^10^. The final K for subsequent analyses was determined by choosing the one that offered the highest stability across runs^10^.

## Selection of the number of latent factors

An important model parameter is the number of latent factors K. Therefore, we determined how factor estimation changed from K = 2 to 10 factors. An exhaustive search was performed to quantify the possibility that two disconnection patterns in the (K+1)-factor model were subdivisions of a disconnection pattern in the K-factor model (while the remaining K-1 disconnection patterns remained similar across both models).

To determine if two disconnection patterns in the (K+1)-factor model were subdivisions of a disconnection pattern in the K-factor model, we averaged the probability distributions of the i-th and j-th latent factors (Pr(Voxel | Factor_i) and Pr(Voxel | Factor_j)) from the (K+1)-factor model to form a single combined probability distribution, Pr(Voxel | Factor_combined). This combined distribution was then compared to the distributions in the K-factor model. Factors in the (K+1)-factor model were reordered using the Hungarian matching algorithm to maximize the correlation with the K-factor model. The pairwise correlations between matched factors were averaged to indicate the quality of the split. By performing an exhaustive search over all pairs of i and j, we found the disconnection factor of the K-factor model whose split best approximated the (K+1)-factor model.

**Supplemental results**

| **Table S1**  Baseline characteristics of members of the Study of Factors Influencing Poststroke Dementia (STROKDEM) cohort excluded from or included in the present study. | | | |
| --- | --- | --- | --- |
|  | **STROKDEM members without follow-up** | **STROKDEM members with follow-up** | **Effect /**  **p-value^a^** |
| **Demographical data** |  |  |  |
| n | 73 | 105 |  |
| Age, years | 66 ± 13 | 63 ± 12 | 2.20 / 0.028 |
| Males, n (%) | 37 (51%) | 72 (69%) | 5.80 / 0.016 |
| Educational level, years | 11 ± 3 | 12 ± 4 | -1.77 / 0.08 |
| **Medical history** |  |  |  |
| IQCODE score | 49 ± 3 | 49 ± 2 | 1.16 / 0.25 |
| Diabetes mellitus, n (%) | 10 (14%) | 13 (12%) | 0.07 / 0.80 |
| Hypertension, n (%) | 43 (59%) | 59 (56%) | 0.13 / 0.72 |
| Body mass index | 27 ± 4 | 27 ± 4 | 0.13 / 0.89 |
| Hyperlipidemia, n (%) | 40 (55%) | 44 (42%) | 2.87 / 0.09 |
| Tobacco use, n (%) | 17 (23%) | 19 (18%) | 0.72 / 0.40 |
| **Admission data** |  |  |  |
| NIHSS score | 1 (0; 2) | 1 (0; 2) | 11.813/ 0.107 |
| WMH volume, cm^3^ | 12.22 ± 17.90 | 9.01 ± 15.83 | 1.97 / 0.06 |
| Infarct volume, cm^3^ | 7.74 ± 11.95 | 10.28 ± 17.10 | 0.36 / 0.72 |
| Left hemisphere stroke, n (%) | 36 (49%) | 56 (53%) | 0.28 / 0.60 |
| Supra-tentorial stroke, n (%) | 69 (95%) | 87 (83%) | 3.41 / 0.05 |
| Affected vascular territories, n of MCA (%) | 49 (67%) | 65 (62%) | 9.824 / 0.365 |
| Abbrevations: IQCODE: Informant Questionnaire on Cognitive Decline in the Elderly; NIHSS: National Institute of Health Stroke Scale; WMH: white matter hyperintensity; MCA: middle cerebral artery.  For affected vascular territories, when an infarct involved multiple vascular territories, we categorized it based on the territory that encompassed the largest portion of the infarct volume.  Quantitative variables are quoted as the mean ± SD or the median (interquartile range).  A χ^2^ test and Wilcoxon rank sum test were applied to categorical and continuous variables, respectively. Uncorrected p values are displayed and P values <0.05 corrected for multiple comparisons are given in bold type.  The effect corresponded to the estimated effect size of each variable on the outcome of interest using χ^2^ or Wilcoxon statistics. | | | |

**Table S2**

Likelihood ratio test results for comparison between logistic regression models

| **Model** | **Log-Likelihood** | **χ^2^** | **p-value** |
| --- | --- | --- | --- |
| **Month 36** | | | |
| Model 1: Cog ~ MOCA + WMH + Factor 1 + Factor 2 + Age + Edu  **p_MOCA_=0.018**; p_WMH_=0.84; p_Factor 1_=0.46 ; **p_Factor 2_=0.046**; p_Age_=0.76; p_Edu_=0.11 ^(1)^ | -55.539 | 2.58 | **0.11** |
| Model 2: Cog ~ MOCA + WMH + Factor 2 + Age + Edu  **p_MOCA_=0.017**; p_WMH_=0.396; **p_Factor 2_=0.006** ; p_Age_=0.618; p_Edu_=0.15 ^(1)^ | -56.829 |  |  |
| Abbreviations: WMH: white matter hyperintensity volume; Factor 1: factor 1 from tract-based disconnection by WMH; Factor 2: factor 2 from tract-based disconnection by WMH; Edu: educational level.  We assumed that the likelihood ratio statistic follows a χ^2^ distribution.  p-value > 0.05 means that Model 1 is not significantly better than Model 2  ^(1)^ p-values of logistic regression model | | | |

**Table S3**

Demographic and clinical data at 36 months poststroke for converter and non-converter participants.

| **Month 36** | | | | | |
| --- | --- | --- | --- | --- | --- |
|  | **Converters** | | **Non-converters** | | **Effect / p-value^a^** |
|  | **COG+** | **COG-** | **COG+** | **COG-** |  |
| **Demographical data** |  |  |  |  |  |
| N | 10 | 25 | 30 | 40 |  |
| Age, years | 65 ± 11 | 64 ± 13 | 66 ± 12 | 59 ± 11 | 6.635 / 0.084 |
| Males, n (%) | 8 (80%) | 18 (72%) | 18 (60%) | 28 (70%) | 1.803 / 0.614 |
| Educational level, years | 11 ± 4 | 12 ± 4 | 10 ± 3 | 13 ± 5 | 10.671 / **0.014** ^(3 < 4)^ |
| **Medical history** |  |  |  |  |  |
| IQCODE score | 49 ± 2 | 49 ± 2 | 49 ± 3 | 48 ± 1 | 2.878 / 0.411 |
| Diabetes mellitus, n (%) | 0 (0%) | 5 (20%) | 4 (13%) | 4 (10%) | 2.985 / 0.394 |
| Hypertension, n (%) | 8 (80%) | 15 (60%) | 19 (63%) | 17 (43%) | 6.118 / 0.106 |
| Body mass index | 28 ± 4 | 28 ± 3 | 28 ± 5 | 26 ± 4 | 4.540 / 0.209 |
| Hyperlipidemia, n (%) | 6 (60%) | 9 (36%) | 16 (53%) | 13 (33%) | 4.766 / 0.190 |
| Tobacco use, n (%) | 2 (20%) | 6 (24%) | 5 (17%) | 6 (15%) | 0.912 / 0.822 |
| **Admission data** |  |  |  |  |  |
| NIHSS score | 1 (0; 1) | 0 (0; 1) | 1 (0; 2) | 0 (0; 1) | 20.285 / 0.161 |
| Fibrinolysis, n (%) | 2 (20%) | 7 (28%) | 9 (30%) | 10 (25%) | 0.378 / 0.945 |
| WMH volume, cm^3^ | 22.53 ± 38.06 | 8.47 ± 9.31 | 10.63 ± 13.72 | 4.76 ± 6.77 | 1.784 / 0.155 |
| Infarct volume, cm^3^ | 12.74 ± 20.02 | 6.84 ± 9.61 | 12.58 ± 22.33 | 9.86 ± 14.97 | 0.647 / 0.586 |
| Left hemisphere stroke, n (%) | 4 (40%) | 12 (48%) | 20 (67%) | 20 (50%) | 3.321 / 0.345 |
| Supra-tentorial stroke, n (%) | 9 (90%) | 22 (88%) | 24 (80%) | 32 (80%) | 1.227 / 0.747 |
| Abbreviations: COG+: participants with cognitive impairment at 6 or 36 months post-stroke; COG-: participants without cognitive impairment at 6 or 36 months post-stroke; IQCODE: Informant Questionnaire on Cognitive Decline in the Elderly; NIHSS: National Institute of Health Stroke Scale; WMH: white matter hyperintensity.  Quantitative variables are quoted as the mean ± standard deviation or the median (interquartile range). Uncorrected P values were displayed and P values <0.05 corrected for multiple comparisons are given in bold type.  ^a^ A χ^2^ test and the Kruskal-Wallis H test were applied to categorical and quantitative variables, respectively. Significant post-hoc test results (Bonferroni correction) are given in brackets. | | | | | |

Given that the three infarct-based latent factors align with the topography of the infarct locations, we tested the association between PSCI status and these infarct-based latent factors, including in each logistic regression model only the patients who were susceptible to express the factor. So, we included 39 patients with right supra-tentorial infarct for factor 1, 48 patients with left supra-tentorial infarct for factor 2, and 18 patients with infra-tentorial infarct for factor 3.

**Table S4**

Associations between infarct-based latent factors and cognitive PSCI status at 6- and 36-month poststroke using subgroups of patients

|  | **Month 6** | | | **Month 36** | | |
| --- | --- | --- | --- | --- | --- | --- |
| Independent variable | β ± SE | OR  [95% CI] | P value | β ± SE | OR  [95% CI] | P value |
| **Model for factor 1** | | | | | | |
| Factor 1 | -3.77 ± 2.60 | 0.02  [1e-5 ; 1.04] | 0.15 | -1.12 ± 1.55 | 0.33  [0.01 ; 5.87] | 0.47 |
| Age | 0.02 ± 0.03 | 1.02  [0.97 ; 1.09] | 0.43 | 0.02 ± 0.03 | 1.02  [0.97 ; 1.09] | 0.42 |
| Educational level | -0.05 ± 0.07 | 0.95  [0.81 ; 1.08] | 0.44 | -0.13 ± 0.08 | 0.87  [0.73 ; 1.01] | 0.10 |
| **Model for factor 2** | | | | | | |
| Factor 2 | 1.74 ± 1.18 | 5.69  [0.63 ; 76.8] | 0.14 | 1.45 ± 1.31 | 4.26  [0.42 ; 96.2] | 0.27 |
| Age | 0.02 ± 0.03 | 1.02  [0.96 ; 1.08] | 0.50 | 0.01 ± 0.03 | 1.01  [0.96 ; 1.07] | 0.69 |
| Educational level | -0.12 ± 0.09 | 0.89  [0.74 ; 1.05] | 0.16 | -0.13 ± 0.09 | 0.87  [0.71 ; 1.04] | 0.15 |
| **Model for factor 3** | | | | | | |
| Factor 3 | -6.15 ± 4.21 | 2e-3  [2e-8 ; 0.81] | 0.14 | -0.62 ± 1.81 | 0.54  [0.01 ; 21.2] | 0.73 |
| Age | -0.05 ± 0.08 | 0.95  [0.77 ; 1.10] | 0.52 | 0.04 ± 0.06 | 1.04  [0.92 ; 1.19] | 0.55 |
| Educational level | -0.84 ± 0.46 | 0.43  [0.12 ; 0.86] | 0.07 | -0.10 ± 0.23 | 0.90  [0.55 ; 1.40] | 0.64 |
| Logistic regression model: Cog ~ Independent Variable + Age + Edu  Abbreviations: SE: standard error; OR: odds ratio; CI: confidence interval; Cog: cognitive status (0 for no PSCI and 1 for PSCI); Edu: educational level (in years)  β is the estimated coefficients for each independent variable, indicating the direction and strength of the relationship with the cognitive status.  SE is the standard error of the coefficient β.  P values <0.05 corrected by FDR are given in bold type. | | | | | | |

For each subject, the white matter mask was segmented on T1-weighted images using VolBrain software (volbrain.net). The normal-appearing white matter (NAWM) masks for both hemispheres were constructed by subtracting the WMH and infarct masks from the white matter masks. Fractional anisotropy (FA), axial diffusivity (AD), and radial diffusivity (RD) were computed from DTI data acquired within 72 hours of admission to the hospital using a 3T MRI scanner (Achieva, Philips, Best, the Netherlands) with a 16-channel neurovascular coil and 16 gradient directions. Median voxel values for FA, AD, and RD were then extracted from contralesional NAWM and WMH, as well as ipsilesional NAWM and infarct. Wilcoxon signed-rank test was used for statistical comparison of diffusivity anisotropy values in NAWM, WMH, and infarct.

**Table S5**

Diffusivity anistropy metrics in normal-appearing white matter, WMH, and infarct

| **Variable, median (IQR)** | **NAWM** | **WMH** | **p-value** | **NAWM** | **Infarct** | **p-value** |
| --- | --- | --- | --- | --- | --- | --- |
| FA | 0.34 (0.32, 0.35) | 0.29 (0.26, 0.32) | **<0.0001** | 0.33 (0.32, 0.35) | 0.29 (0.24, 0.33) | **<0.0001** |
| AD (x10^-3^), mm^2^/s | 1.07 (1.04, 1.09) | 1.34 (1.27, 1.39) | **<0.0001** | 1.07 (1.05, 1.09) | 1.20 (1.13, 1.27) | **<0.0001** |
| RD (x10^-3^), mm^2^/s | 0.63 (0.61, 0.64) | 0.82 (0.78, 0.87) | **<0.0001** | 0.64 (0.61, 0.66) | 0.75 (0.69, 0.82) | **<0.0001** |
| Abbrevations: WMH: white matter hyperintensity; NAWM: normal-appearing white matter ; FA: fractional anisotropy ; AD: axial diffusivity ; RD: radial diffusivity.  Variables are quoted as the median (interquartile range).  A Wilcoxon signed-rank test were applied and p-values < 0.05 corrected for multiple comparisons are given in bold type. | | | | | | |

| 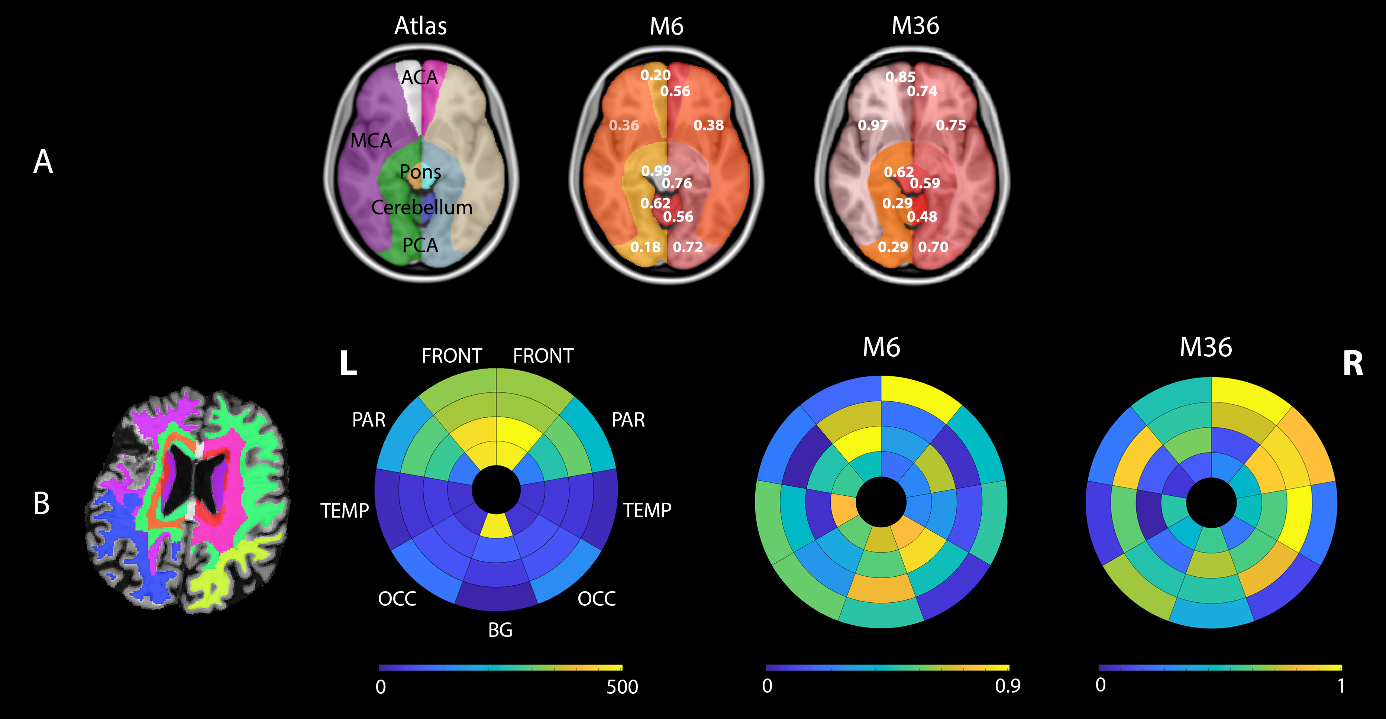 |
| --- |
| **Figure S1:** (A) Infarct volumes by vascular territories. From left to right: the atlas of vascular territories, statistical difference between participants with and without 6 months PSCI (color scale: 1 – p-value and p-value in white text), statistical difference between participants with and without 36 months PSCI (color scale: 1 – p-value and p-value in white text). No significant difference at p<0.05 FDR corrected.  (B) Regional patterns of WMH. From left to right: an example of lobar segmentations, bullseye representation of WMH load for all participants, bullseye representation of the statistical difference between participants with and without 6 months PSCI (color scale: 1 – p-value), bullseye representation of the statistical difference between the 4 groups at 36 months PSCI (color scale: 1 – p-value). No significant difference at p<0.05 FDR corrected.  Abbreviations: MCA: middle cerebral artery; PCA: posterior cerebral artery; ACA: anterior cerebral artery, BG basal ganglia, FRONT frontal lobe, PAR parietal lobe, OCC occipital lobe, TEMP temporal lobe |

**References**

1. Golden, C. J. *Stroop Color and Word Test: A Manual for Clinical and Experimental Uses.* (Wood Dale, IL., 2002).

2. Amieva, H. *et al.* Trail Making Test: normative data in a French elderly population from the three Cities Study. *Rev. Neuropsychol.* **1**, 210–220 (2009).

3. Osterrieth, P. A. Le test de copie d’une figure complexe; contribution à l’étude de la perception et de la mémoire. [Test of copying a complex figure; contribution to the study of perception and memory.]. *Arch. Psychol.* **30**, 206–356 (1944).

4. Grober, E. & Buschke, H. Genuine memory deficits in dementia. *Dev. Neuropsychol.* **3**, 13–36 (1987).

5. Deloche, G. & Hannequin, D. *Test de denomination orale d’images: DO 80*. (Editions du Centre de psychologie appliquee, Paris, 1997).

6. Cardebat, D., Doyon, B., Puel, M., Goulet, P. & Joanette, Y. [Formal and semantic lexical evocation in normal subjects. Performance and dynamics of production as a function of sex, age and educational level]. *Acta Neurol. Belg.* **90**, 207–217 (1990).

7. Quental, N. B. M., Brucki, S. M. D. & Bueno, O. F. A. Visuospatial function in early Alzheimer’s disease--the use of the Visual Object and Space Perception (VOSP) battery. *PloS One* **8**, e68398 (2013).

8. Schirmer, M. D. *et al.* Spatial Signature of White Matter Hyperintensities in Stroke Patients. *Front. Neurol.* **10**, (2019).

9. Jiménez-Balado, J., Corlier, F., Habeck, C., Stern, Y. & Eich, T. Effects of white matter hyperintensities distribution and clustering on late-life cognitive impairment. *Sci. Rep.* **12**, 1955 (2022).

10. Zhang, X. *et al.* Bayesian model reveals latent atrophy factors with dissociable cognitive trajectories in Alzheimer’s disease. *Proc. Natl. Acad. Sci. U. S. A.* **113**, E6535–E6544 (2016).

11. Blei, D. M., Ng, A. Y. & Jordan, M. I. Latent dirichlet allocation. *J. Mach. Learn. Res.* **3**, 993–1022 (2003).
